# Comparison of long-term outcomes between endoscopic submucosal dissection and esophagectomy for superficial esophageal squamous cell carcinoma

**DOI:** 10.1093/gastro/goag032

**Published:** 2026-04-19

**Authors:** Byeong Yun Ahn, Quanxin Zheng, Soo-Jeong Cho, Sang Gyun Kim, Chang Hyun Kang, Hyunsoo Chung

**Affiliations:** Department of Internal Medicine & Liver Research Institute, Seoul National University College of Medicine, Seoul, South Korea; Department of Internal Medicine & Liver Research Institute, Seoul National University College of Medicine, Seoul, South Korea; Department of Internal Medicine & Liver Research Institute, Seoul National University College of Medicine, Seoul, South Korea; Department of Internal Medicine & Liver Research Institute, Seoul National University College of Medicine, Seoul, South Korea; Department of Thoracic and Cardiovascular Surgery, Seoul National University Hospital, Seoul National University College of Medicine, Seoul, South Korea; Department of Internal Medicine & Liver Research Institute, Seoul National University College of Medicine, Seoul, South Korea; Department of Medical Device Development, Seoul National University College of Medicine, Seoul, South Korea

**Keywords:** esophageal squamous cell carcinoma, endoscopy, gastrointestinal, esophagectomy, treatment outcome, propensity score

## Abstract

**Background:**

Endoscopic submucosal dissection (ESD) is increasingly being performed as a less invasive alternative to esophagectomy for superficial esophageal squamous cell carcinoma (SESCC); however, comparative long-term outcome data, especially for non-curative resection (non-CR) cases and elderly patients, remain limited. This study aimed to compare the clinical outcomes of ESD and esophagectomy using a propensity score (PS)–matched cohort.

**Methods:**

Patients with SESCC who underwent ESD or esophagectomy at a tertiary referral center between 2011 and 2021 were retrospectively reviewed. In the PS-matched cohort, overall survival (OS), disease-specific survival (DSS), recurrence-free survival (RFS), additional treatments, and adverse events were compared. Subgroup analyses were conducted for non-CR cases and elderly patients (≥70 years).

**Results:**

Among the 63 PS-matched pairs, OS, DSS, and RFS were comparable between the ESD and esophagectomy groups. The 5-year OS, DSS, and RFS rates were 89.9% versus 79.2%, 95.7% versus 94.4%, and 90.6% versus 89.1%, respectively. The ESD group had significantly fewer adverse events (47.6% vs 68.3%, *P *< 0.05) and a shorter median hospital stay (1.0 vs 10.0 days, *P *< 0.001) than the esophagectomy group. Survival outcomes were also similar in the non-CR and elderly subgroups.

**Conclusions:**

ESD is a safe and effective alternative to esophagectomy for SESCC, including in non-CR and elderly patients. Given the limitations of preprocedural depth assessment and the high risk of complications associated with esophagectomy, staging ESD for SESCC may represent a reasonable treatment option, particularly for elderly or high-risk patients.

## Introduction

Esophageal cancer remains a significant global health concern, ranking eighth in terms of incidence and sixth in terms of cancer-related mortality worldwide [1]. Esophageal squamous cell carcinoma (ESCC), a major histological subtype of esophageal cancer, has a potentially improved prognosis when detected at an early stage [2]. Superficial ESCC (SESCC) refers to early-stage ESCC, in which tumor invasion is limited to the epithelium (EP; Tis), lamina propria, muscularis mucosa (LP/MM; T1a), or submucosa (SM; T1b) [3]. The treatment options for SESCC, including surgical resection, chemoradiotherapy (CRT), and endoscopic resections, are primarily determined based on the depth of the invading tumor, which is closely associated with the risk of lymph node (LN) metastasis [4]. Current clinical guidelines recommend endoscopic submucosal dissection (ESD) as a choice for the treatment of SESCC when the risk of LN is negligible, such as in non-circumferential, mucosa-confined ESCC [4–7].

ESD has been increasingly utilized as a less-invasive alternative to surgery, with comparable cancer-specific outcomes for the management of SESCC [8–11]. Recent guidelines from Japan and Europe recommend ESD for lesions clinically staged as MM/SM1, defined as tumor invasion into the MM or the superficial SM (<200 μm) [4, 5]. This expansion of indications is partly driven by the risk of over-staging owing to the limited accuracy of preoperative depth assessments, which may result in unnecessary surgical resection [12, 13]. Furthermore, even in cases of non-curative resection (non-CR), defined by SM or lymphovascular (LV) invasion, or a positive vertical resection margin on ESD pathology, favorable outcomes can be achieved with appropriate additional treatments [14]. Therefore, in patients with SESCC who are expected to achieve non-CR, staging ESD may be a viable treatment option, especially for those at high surgical risk.

However, several high-quality real-world cohort studies have compared ESD with esophagectomy for early-stage esophageal squamous cell carcinoma and have reported comparable long-term survival and lower perioperative morbidity in the ESD group [9, 11]. In addition, a recent multicenter study of ESD for pathologically stage T1b esophageal cancer highlighted the limited accuracy of preprocedural endoscopic ultrasound (EUS) and proposed “staging ESD” as an initial therapeutic and staging approach. Notably, this previous study did not include a surgical control group and focused primarily on technical and recurrence outcomes after ESD [13]. Despite these important contributions, previous studies have provided limited data on non-CR cases, details of additional treatments after the initial procedure, and outcomes in elderly patients at high surgical risk within an ESD-based treatment pathway [9, 13, 15, 16]. This study aimed to compare the long-term clinical outcomes of patients with SESCC who underwent ESD or esophagectomy, with a focus on the non-CR and elderly subgroups.

## Patients and methods

### Study population

This study retrospectively analyzed patients with SESCC who underwent ESD or esophagectomy at the Seoul National University Hospital, Korea. Among the patients who were treated with esophageal ESD between 2011 and 2021, those with (i) multiple primary cancers at diagnosis; (ii) prior CRT, endoscopic treatment, or esophagectomy; (iii) other types of neoplasms; and (iv) low-grade dysplasia were excluded from the analysis. Among the patients who underwent esophagectomy for ESCC between 2014 and 2021, exclusions were made for (i) multiple primary cancers at diagnosis, (ii) prior CRT, endoscopic treatment, esophagectomy, or rescue operation after ESD, (iii) other types of neoplasms, and (iv) invasion of the muscularis propria or LN metastasis. All ESD and esophagectomy procedures in this study were performed with curative intent in patients deemed suitable for definitive treatment. Patients who underwent ESD or surgery as purely palliative treatment were not included.

### Pretreatment evaluation

All patients in both cohorts underwent a comprehensive preoperative evaluation, including endoscopy (chromoendoscopy with Lugol’s solution and/or narrow-band imaging), EUS, and chest and abdominal computed tomography (CT). EUS was used to assess tumor depth and detect mediastinal LN metastases. Chest and abdominal CT were performed to evaluate regional LN involvement and distant metastases. When LN metastasis was suspected on CT, positron emission tomography was additionally performed for further assessment. Patients with overt regional lymph node or distant metastasis on imaging were not considered candidates for ESD and were excluded from the present analysis; therefore, only patients who were clinically node-negative (cN0) were included in the ESD group, whereas patients who were pathologically node-negative (pN0) were included in the esophagectomy group.

### Procedure and histologic evaluation

Experienced endoscopists performed esophageal ESD under general anesthesia using a standard single-channel endoscope (GIF-H260 or GIF-HQ290; Olympus Corp., Tokyo, Japan) and an insulated tip knife (ITknife nano, KD-612U; Olympus Corp.) [17]. The tumor margins were delineated by using 2% Lugol’s solution or image-enhanced endoscopy, and marking dots were placed 2–3 mm beyond the tumor boundary by using an electrosurgical knife. Following the SM injection of a solution of normal saline mixed with indigo carmine, circumferential mucosal precutting and SM dissection were performed. The resected specimens were carefully stretched and pinned onto a board. After fixation in 10% formalin and staining with hematoxylin and eosin, histopathological evaluation was conducted on 2-mm-thick sections. In the esophagectomy group, the Ivor Lewis or McKeown procedure was primarily performed, whereas transhiatal esophagectomy was performed in selected cases. Following routine fixation, surgical specimens were sectioned at 4-mm thickness for evaluation.

In both groups, the pathological specimens were analyzed for tumor histology, differentiation, size, depth of invasion, LV invasion, and involvement of the resection margins. Mediastinal LN metastases were pathologically evaluated only in surgical specimens. Tumor staging was performed according to the eighth edition of the American Joint Committee on Cancer classification for esophageal and esophagogastric junction malignancies [18]. In accordance with the Japan Esophageal Society guidelines, the depth of SM invasion was classified into SM1 (≤200 µm from the MM) and SM2 (>200 µm from the MM) [3]. For ESD specimens, CR was defined as tumor resection with negative vertical resection margins and no evidence of SM or LV invasion on microscopic examination. Non-CR was defined as the presence of at least one risk factor for recurrence: SM or LV invasion or a positive vertical resection margin on ESD pathology [6, 7]. In patients with two primary esophageal lesions, the one with the deeper level of invasion was selected for analysis.

### Additional treatments and follow-up

In the ESD group, the final decision regarding additional treatment after non-CR on the final ESD pathology was determined through multidisciplinary team (MDT) discussions. Treatment decisions were made during regular MDT meetings held every 1–2 weeks. The MDT included specialists in thoracic surgery, gastroenterology, radiology, pathology, medical oncology, and radiation oncology. During these meetings, complex or uncertain cases were discussed in detail, and treatment decisions were determined by comprehensively integrating the patient’s clinical condition, age, personal preference, and tumor-related factors. Based on this discussion, additional treatment options included surgery or radiotherapy (RT; 45 Gy in 25 fractions). Endoscopic therapies, including argon plasma coagulation, were selectively performed in patients with positive lateral resection margins. In the esophagectomy group, RT or CRT therapy was advised when tumor involvement in the surgical resection margin was identified. Patients deemed unfit for further treatment due to advanced age or significant comorbidities were followed up at 6-month intervals.

Post-treatment surveillance for recurrence was intensively conducted. In both the ESD and esophagectomy groups, chest CT was performed every 6 months during the first 2 years, and annually thereafter for up to 5 years. In the ESD group, endoscopic evaluations were performed at 6 months, 1 year, 18 months, and 2 years, and then annually thereafter. In the esophagectomy group, endoscopy was performed annually after treatment. To evaluate recurrent laryngeal nerve injury, nasal laryngoscopy was performed on postoperative day 3 to assess vocal cord function in the esophagectomy group. Follow-up data were primarily obtained from medical records.

### Variables and outcomes

The variables included age, sex, Charlson Comorbidity Index (CCI) score, preoperative depth assessment by EUS, and tumor pathology, including location, size, macroscopic type, histologic subtype, differentiation grade, circumferential involvement, LV invasion, resection margin status, and depth of invasion. Regional LN involvement was assessed in the esophagectomy group. Other variables included the length of hospital stay, adverse events, follow-up duration, additional treatments received, pattern of cancer recurrence, and cause of death.

Overall survival (OS) was defined as the time interval from ESD or esophagectomy to death from any cause. Disease-specific survival (DSS) was defined as the time from the procedure to death attributable to cancer recurrence. Recurrence-free survival (RFS) was defined as the time from the primary procedure (or the additional procedure, if performed) to the occurrence of distant or LN metastasis. In the RFS analysis, patients who died without evidence of cancer recurrence were censored at the time of their last follow-up visit. Additionally, patients diagnosed with local recurrence, metachronous recurrence, or a second primary malignancy were censored on the date of diagnosis of the respective lesion. Metachronous recurrence was defined as the development of tumors in the esophagus, other than those at the primary resection site, one year after ESD.

Adverse events were assessed as early (within 1 month post-procedure), late (beyond 1 month post-procedure), or additional treatment-related, and their severity was classified by using the Clavien-Dindo system: Grade I (requiring observation only), Grade II (requiring transfusion or pharmacologic therapy), Grade III (requiring surgical, endoscopic, or radiologic intervention), Grade IV (requiring intensive care), and Grade V (death). In the ESD group, adverse events included perforation, bleeding requiring transfusion, and strictures necessitating intervention. Postoperative adverse events in the esophagectomy group included pulmonary complications (respiratory insufficiency and pneumonia), cardiovascular events (arrhythmia), renal events (acute kidney injury), and surgical complications, such as vocal cord paralysis, wound infection or dehiscence, anastomotic leakage, edematous pyloric narrowing, chyle leakage, bleeding, fistula formation, stricture, and recurrent ileus.

### Statistical analysis

Continuous variables were compared by using Student’s *t*-test or the Mann–Whitney *U* test, as appropriate, based on data distribution, whereas categorical variables were analyzed by using the chi-square test or Fisher’s exact test, as applicable.

To minimize selection bias, propensity score (PS) matching was performed by using a 1:1 nearest-neighbor algorithm with a caliper width of 0.2. PS was estimated by using a multivariable logistic regression model that included the following covariates: age, sex, CCI, BMI, tumor location, macroscopic type, tumor size, circumferential extent, differentiation grade, invasion depth, and LV invasion. Tumor involvement at the resection margin was excluded from the matching process because a positive resection margin in radical surgery indicates more diffuse disease than the same condition in ESD. Additional treatment was also excluded as a covariate because of the differing indications for the two treatment modalities. After matching, the covariate balance between the groups was assessed using standardized mean differences, with an absolute standardized mean difference of less than 0.1 considered indicative of adequate balance. The matched cohort was then used to compare the OS, DSS, and RFS between the ESD and esophagectomy groups. Kaplan–Meier survival curves were constructed for OS, DSS, and RFS, and group differences were evaluated by using the log-rank test.

Two subgroup analyses were conducted. PSs were estimated for the entire study population by using the same covariates as those used in the main analysis. A 1:1 PS matching was performed between patients in the non-CR subgroup of the ESD group and those in the esophagectomy group who met the non-CR criteria defined for ESD (i.e. tumors with positive vertical resection margins, mucosal cancer with lymphovascular invasion, or submucosal cancer). These criteria were applied as a theoretical, non-CR–equivalent classification for comparative purposes, because all patients in the esophagectomy group underwent definitive primary surgery. Survival outcomes were subsequently compared between the matched subgroups. In the second subgroup analysis, elderly patients were defined as those aged ≥69 years, based on previous reports that used age thresholds around 66–85 years and the age distribution of the cohort of the present study [19–21]. Patients aged ≥69 years from both treatment groups, with 1:1 PS matching, were included prior to the comparison of survival outcomes.

Statistical significance was set at a two-sided *P* value < 0.05. All statistical analyses were performed using the R software (version 3.4.1; R Foundation for Statistical Computing, Vienna, Austria).

### Ethical approval

This study was conducted in accordance with the ethical standards of the Declaration of Helsinki. This study was approved by the Institutional Review Board of the Seoul National University Hospital (IRB No. H-2407-083-1553; approved on September 23, 2024). Owing to the retrospective nature of the study, the need for informed consent was waived.

## Results

### Baseline characteristics of all patients and the matched cohort

**Figure 1:**
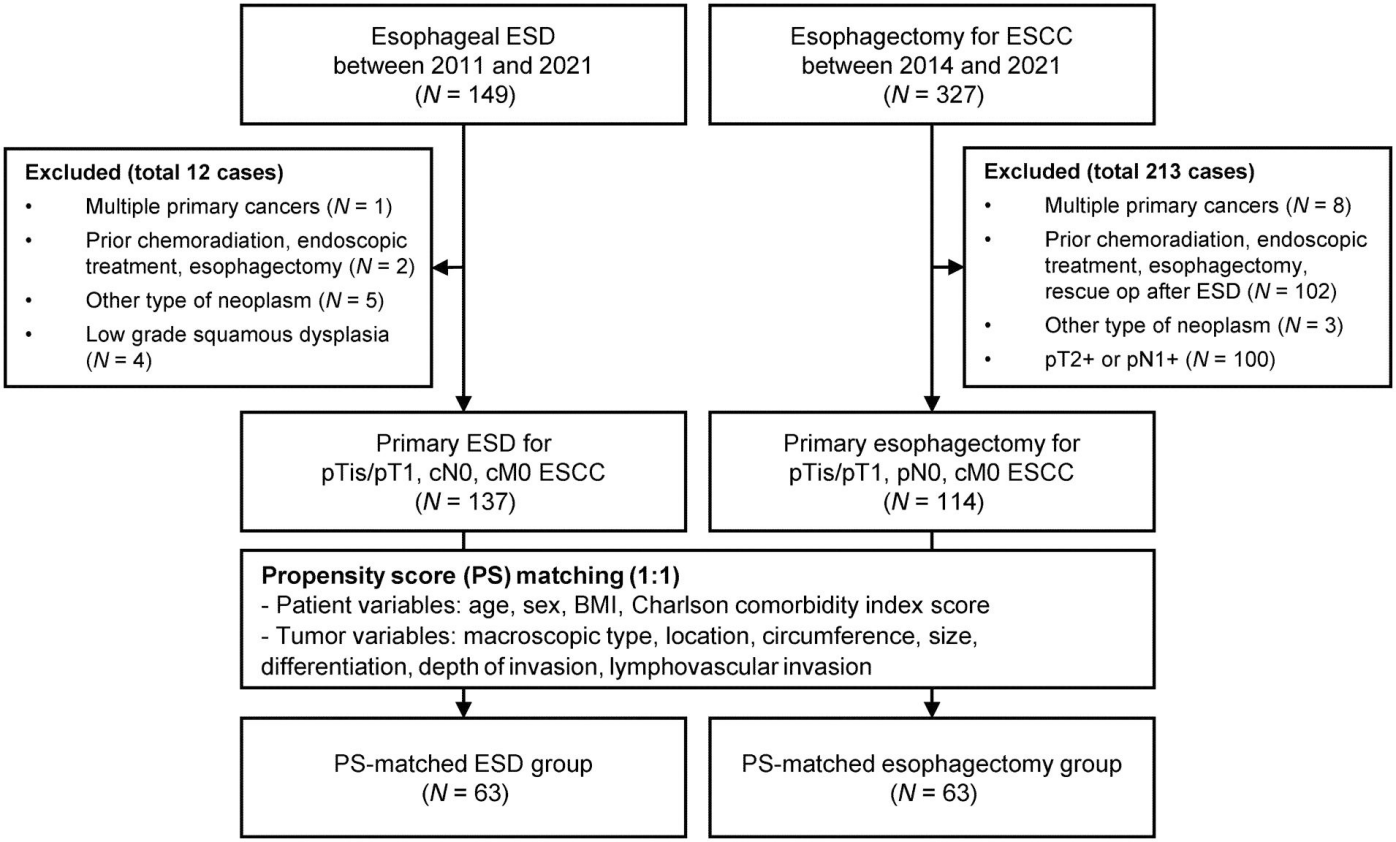
Flowchart of study population selection. ESD, endoscopic submucosal dissection; ESCC, esophageal squamous cell carcinoma.

Among the 251 patients included in the study, the ESD group comprised 137 patients with pTis–pT1, cN0, and cM0 ESCC, whereas the esophagectomy group included 114 patients with pTis–pT1, pN0, and cM0 ESCC (Fig. 1). All patients underwent either ESD or esophagectomy for a single ESCC, except for one patient who underwent ESD for two lesions and five patients who underwent esophagectomy for two lesions. Lesion-based analyses were performed by using the patient’s most advanced lesion. Before PS matching, the ESD group demonstrated a significantly smaller tumor size, narrower circumferential involvement, shallower clinical and pathological invasion depth, and a higher proportion of well-differentiated tumors than the esophagectomy group. Among the 63 PS-matched pairs, no significant differences were observed between the two groups regarding the covariates used for PS estimation (Table 1).

**Table 1:** Patient and lesion characteristics of the study population

	Cohort before PS matching			Cohort after PS matching		
Characteristic	ESD	Esophagectomy	*P* value	ESD	Esophagectomy	*P* value
	(*N *= 137)	(*N *= 114)		(*N *= 63)	(*N *= 63)	
Age, years	66.7 ± 8.4	67.2 ± 7.4	0.608	66.3 ± 8.2	66.5 ± 7.5	0.910
Male	124 (90.5)	102 (89.5)	0.951	55 (87.3)	58 (92.1)	0.558
BMI, kg/m^2^	23.9 ± 3.3	24.2 ± 3.2	0.498	24.5 ± 2.9	24.6 ± 3.3	0.818
CCI ≥ 2	14 (10.2)	13 (11.4)	0.923	6 (9.5)	7 (11.1)	1.000
Clinical T staging			<0.001			<0.001
cT1a	90 (65.7)	25 (21.9)		45 (71.4)	14 (22.2)	
cT1b	4 (2.9)	65 (57.0)		3 (4.8)	37 (58.7)	
cT2 or over	0 (0.0)	19 (16.7)		0 (0.0)	9 (14.3)	
Not done	43 (31.4)	5 (4.4)		15 (23.8)	3 (4.8)	
Location			0.772			0.898
Cervical or upper thoracic	7 (5.1)	8 (7.0)		3 (4.8)	2 (3.2)	
Middle thoracic	56 (40.9)	48 (42.1)		25 (39.7)	25 (39.7)	
Lower thoracic or abdominal	74 (54.0)	58 (50.9)		35 (55.6)	36 (57.1)	
Macroscopic type			0.086			1.000
Non-flat	11 (8.0)	18 (15.8)		8 (12.7)	9 (14.3)	
Flat	126 (92.0)	96 (84.2)		55 (87.3)	54 (85.7)	
Circumference of tumor			0.026			0.980
<1/4	56 (40.9)	31 (27.2)		20 (31.7)	19 (30.2)	
1/4≤, <1/2	56 (40.9)	46 (40.4)		29 (46.0)	28 (44.4)	
1/2≤, <3/4	11 (8.0)	12 (10.5)		5 (7.9)	6 (9.5)	
3/4≤ or whole circumferential	14 (10.2)	25 (21.9)		9 (14.3)	10 (15.9)	
Differentiation			<0.001			1.000
Well	94 (68.6)	34 (29.8)		26 (41.3)	27 (42.9)	
Moderate to poor	43 (31.4)	80 (70.2)		37 (58.7)	36 (57.1)	
Tumor size, mm	19.0 ± 10.3	29.9 ± 17.0	<0.001	22.5 ± 11.4	25.6 ± 16.3	0.220
Pathologic invasion depth			<0.001			0.922
pTis	57 (41.6)	11 (9.6)		10 (15.9)	11 (17.5)	
pT1a-LP	40 (29.2)	32 (28.1)		25 (39.7)	20 (31.7)	
pT1a-MM	16 (11.7)	11 (9.6)		8 (12.7)	9 (14.3)	
pT1b-SM1 (≤200 μm)	6 (4.4)	7 (6.1)		4 (6.3)	4 (6.3)	
pT1b-SM2 (>200 μm)	18 (13.1)	53 (46.5)		16 (25.4)	19 (30.2)	
Positive LV invasion	13 (9.5)	12 (10.5)	0.951	6 (9.5)	7 (11.1)	1.000
Resection margin			0.183			0.127
Positive lateral margin	1 (0.7)	1 (0.9)		1 (1.6)	1 (1.6)	
Positive vertical margin	4 (2.9)	0 (0.0)		4 (6.3)	0 (0.0)	

Data are presented as mean ± standard deviation or number (%).

PS, propensity score; ESD, endoscopic submucosal dissection; CCI, Charlson Comorbidity Index; LP, lamina propria; MM, muscularis mucosa; SM, submucosa; LV, lymphovascular.

Among the entire study population, preoperative EUS was performed on 203 patients (Table 1). Of the 126 patients with pT1a ESCC, 34 (27.0%) were clinically over-staged, with T1b or deeper invasion. The overall accuracy of EUS in distinguishing T1a from T1b tumors was 71.2% (Supplementary Table S1).

### Follow-up and survival outcomes of the matched cohort

Among the 63 patients in the ESD group, 4 with a positive vertical resection margin and 17 with SM invasion were classified as non-CR. Of these, 9 patients (14.3%) underwent additional surgery, and 11 (17.5%) received RT. One elderly patient, aged 84 years, did not receive any additional treatment. No patients in the ESD group received CRT during the study period. In the esophagectomy group, one patient with R0 resection but a tumor located very close to the resection margin received additional RT. In addition, one patient underwent CRT due to a positive lateral resection margin (Table 2).

**Table 2:** Additional treatments and follow-up of the study population

	Cohort before PS matching			Cohort after PS matching		
Variable	ESD	Esophagectomy	*P* value	ESD	Esophagectomy	*P* value
	(*N *= 137)	(*N *= 114)		(*N *= 63)	(*N *= 63)	
Follow-up duration, months	58.0 (40.0–79.0)	52.5 (32.0–78.0)	0.168	59.0 (40.5–79.0)	54.0 (34.5–86.5)	0.493
Non-CR criteria for ESD			<0.001			0.111
Positive vertical margin	4 (2.9)	0 (0.0)		4 (6.3)	0 (0.0)	
Mucosal cancer with LV invasion	5 (3.6)	1 (0.9)		0 (0.0)	1 (1.6)	
Submucosal invasion	21 (15.3)	60 (52.6)		17 (27.0)	23 (36.5)	
Additional treatment			<0.001			<0.001
Observation	105 (76.6)	111 (97.4)		41 (66.1)	59 (95.2)	
Endoscopic therapy	4 (2.9)	0 (0.0)		2 (3.2)	0 (0.0)	
Surgery	11 (8.0)	0 (0.0)		9 (14.3)	0 (0.0)	
Radiation therapy	17 (12.4)	1 (0.9)		11 (17.5)	1 (1.6)	
Chemoradiation therapy	0 (0.0)	2 (1.8)		0 (0.0)	1 (1.6)	
Primary cancer recurrence			0.259			0.927
Local recurrence	0 (0.0)	1 (0.9)		0 (0.0)	0 (0.0)	
LN metastasis	4 (2.9)	7 (6.1)		3 (4.8)	4 (6.3)	
Distant metastasis	2 (1.5)	4 (3.5)		2 (3.2)	2 (3.2)	
Metachronous recurrence	8 (5.8)	0 (0.0)	0.024	2 (3.2)	0 (0.0)	0.476
Death from any cause	19 (13.9)	25 (21.9)	0.132	6 (9.5)	13 (20.6)	0.135
Death from cancer recurrence	2 (1.5)	4 (3.5)	0.520	2 (3.2)	3 (4.8)	1.000

Data are presented as number (%) or median (interquartile range).

PS, propensity score; ESD, endoscopic submucosal dissection; non-CR, non-curative resection; LN, lymph node.

During the median follow-up periods of 59.0 months for the ESD group and 54.0 months for the esophagectomy group, five and six cases of primary cancer recurrence were observed, respectively. In the ESD group, two metachronous recurrences were detected during surveillance endoscopy. Among the six deaths in the ESD group, two were due to cancer recurrence. In the esophagectomy group, 3 out of 13 deaths were attributed to cancer recurrence (Table 2). Two deaths in the esophagectomy group were associated with surgical complications (Supplementary Fig. S1).

**Figure 2:**
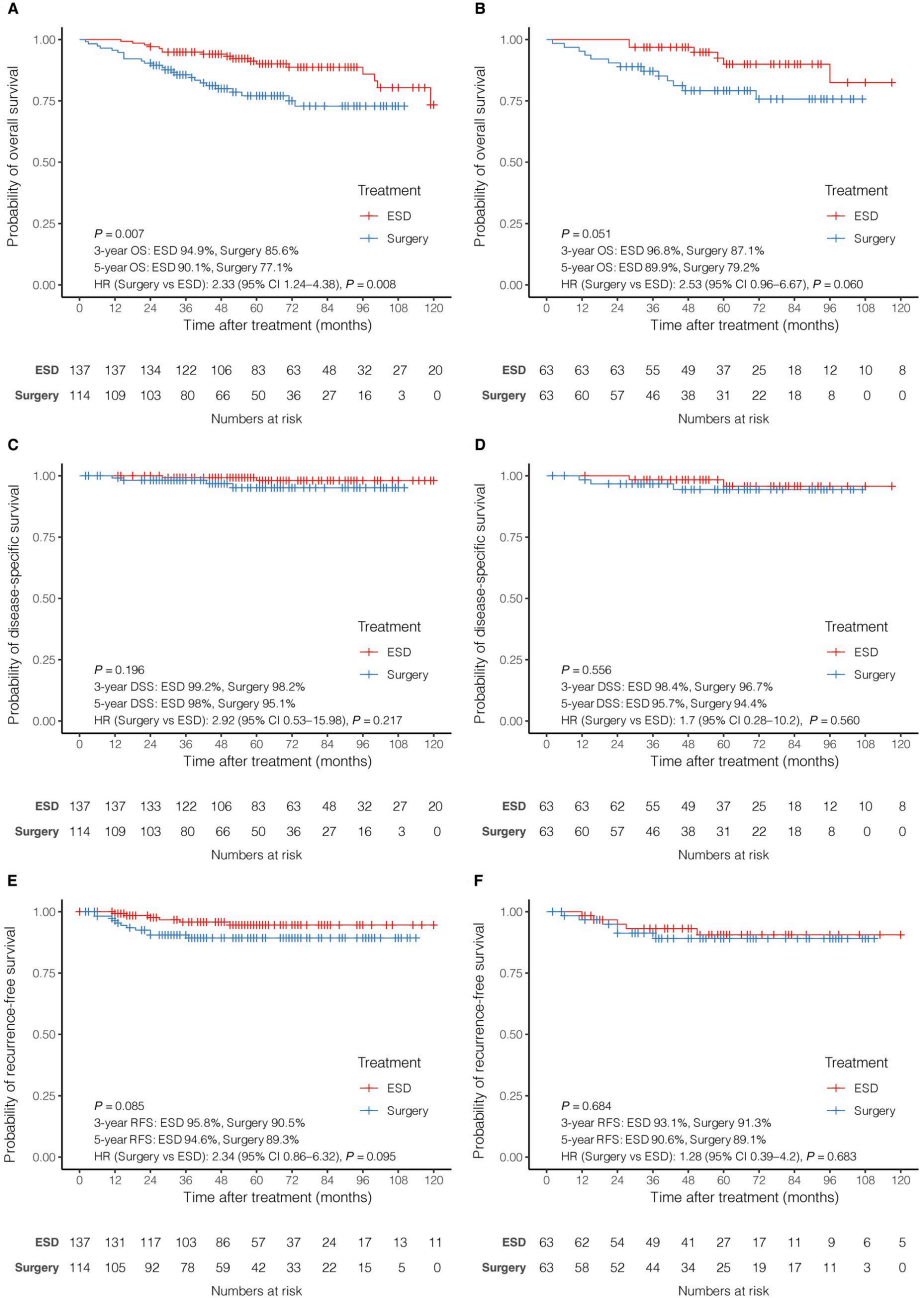
Kaplan-Meier survival curves of the ESD and esophagectomy groups. (A) OS in all patients; (B) OS in the PS-matched cohort; (C) DSS in all patients; (D) DSS in the PS-matched cohort; (E) RFS in all patients; and (F) RFS in the PS-matched cohort. *P* values were calculated by using log-rank tests. OS, overall survival; DSS, disease-specific survival; RFS, recurrence-free survival; ESD, endoscopic submucosal dissection.

In the PS-matched cohort, the 3-year OS rates were 96.8% and 87.1% in the ESD and esophagectomy groups, respectively, and the 5-year OS rates were 89.9% and 79.2%, respectively. A significant difference in OS between the two groups was observed prior to PS matching (*P *= 0.007; Fig. 2A), whereas the difference was not statistically significant after PS matching (*P *= 0.051; Fig. 2B). The 3-year DSS rates were 98.4% and 96.7% in the ESD and esophagectomy groups, respectively, whereas the 5-year DSS rates were 95.7% and 94.4% in the ESD and esophagectomy groups, respectively. The 3-year RFS rates were 93.1% and 91.3% in the ESD and esophagectomy groups, respectively, whereas the 5-year RFS rates were 90.6% and 89.1%, respectively. There were no significant differences in DSS or RFS between the two groups before or after PS matching (Fig. 2C–F).

### Adverse events of a matched cohort

Table 3 summarizes the lengths of hospital stay and adverse events associated with each treatment modality. The median hospitalization duration was significantly shorter in the ESD group than in the esophagectomy group (1.0 vs 10.0 days, *P *< 0.001). The overall and early adverse event rates were also significantly lower in the ESD group than in the esophagectomy group (47.6% vs 68.3%, *P *= 0.010, and 20.6% vs 58.7%, *P *< 0.001, respectively), whereas the rate of additional treatment-related adverse events was higher in the ESD group than in the esophagectomy group (20.6% vs 1.6%, *P = *0.002) (Table 3). In contrast, late adverse event rates did not differ significantly between the two groups, with stricture being the most common late complication in both groups. Severe adverse events (Grade IV–V) were observed only in the esophagectomy group, where one patient required intensive care due to postoperative pneumonia, and two patients died from complications following anastomotic leakage and pneumatosis intestinalis, respectively. No severe adverse events were observed in the ESD group.

**Table 3:** Adverse events of the study population

	Cohort before PS matching			Cohort after PS matching		
Variable	ESD	Esophagectomy	*P* value	ESD	Esophagectomy	*P* value
	(*N *= 137)	(*N *= 114)		(*N *= 63)	(*N *= 63)	
Hospital stay, days	1.0 (1.0–2.0)	11.0 (8.0–15.0)	<0.001	1.0 (1.0–2.0)	10.0 (8.0–13.5)	<0.001
60-day mortality	0 (0.0)	1 (0.9)	1.000	0 (0.0)	1 (1.6)	1.000
Overall adverse events			<0.001		43 (68.3)	0.010
Grade I–II	48 (35.0)	79 (69.3)		30 (47.6)	28 (44.4)	
Grade III	21 (15.3)	50 (43.9)		14 (22.2)	12 (19.0)	
Grade IV–V	27 (19.7) 0 (0.0)	25 (21.9) 4 (3.5)		16 (25.4) 0 (0.0)	3 (4.8)	
Early adverse events (<1 month after treatment)	21 (15.3)	67 (58.8)	<0.001	13 (20.6) *Microperforation 9* *Stricture 6*	37 (58.7) *Pulmonary 8* *Cardiovascular 8* *Renal 1* *Vocal cord palsy 18* *Wound problem 1* *Anastomotic leak 2* *Chyle leakage 4* *Bleeding 1* *Fistula 2* *Ileus 2*	<0.001
Late adverse events (>1 month after treatment)	12 (8.8)	21 (18.4)	0.039	8 (12.7) *Stricture 8*	10 (15.9) *Stricture 6* *Ileus 4*	0.799
Additional treatment-related adverse events	20 (14.6)	2 (1.8%)	<0.001	13 (20.6) *Surgery 8* *Radiation 5*	1 (1.6) *Radiation 1*	0.002

Data are presented as number (%) or median (interquartile range). The severity of adverse events was classified according to the Clavien-Dindo classification.

PS, propensity score; ESD, endoscopic submucosal dissection.

### Survival outcomes of the non-CR cohort

**Figure 3:**
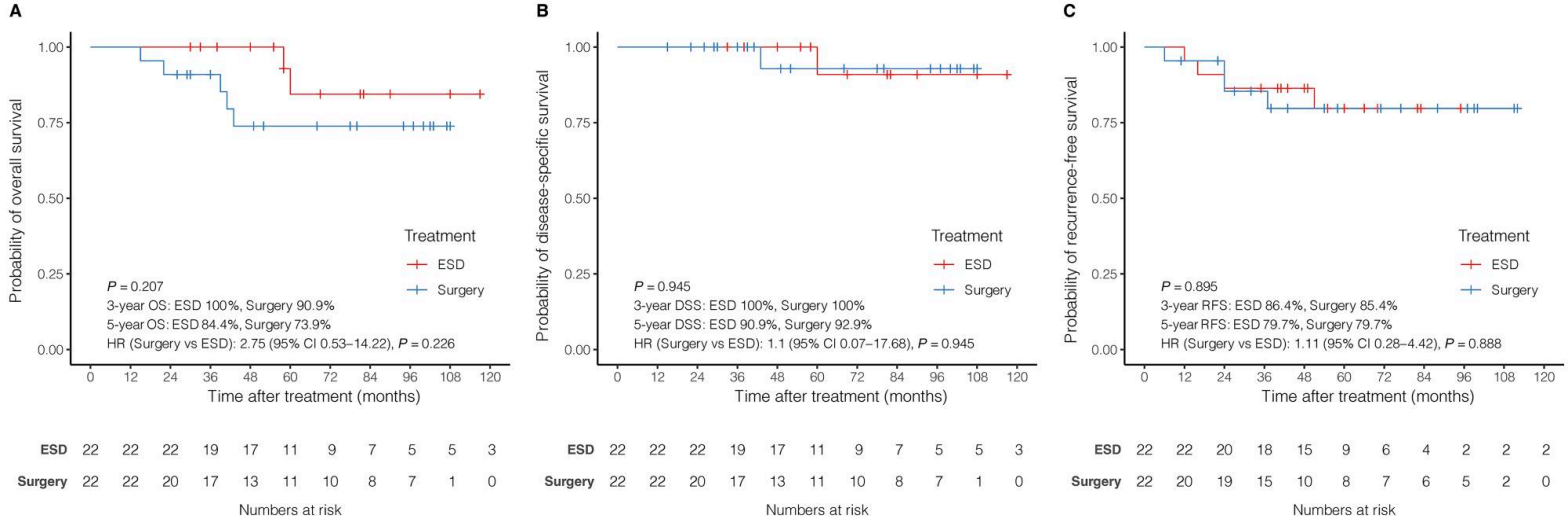
Kaplan-Meier survival curves of the ESD and esophagectomy groups in the non-CR cohort. (A) OS of the non-CR cohort; (B) DSS of the non-CR cohort; and (C) RFS of the non-CR cohort. OS, overall survival; DSS, disease-specific survival; RFS, recurrence-free survival; ESD, endoscopic submucosal dissection.

Before PS matching, 30 patients in the ESD group were classified as having non-CR pathology, including 4 with a positive vertical margin, 5 with mucosal cancer with LV invasion, and 21 with submucosal cancer. According to the same non-CR criteria, the esophagectomy group included 1 patient with mucosal cancer with LV invasion and 60 patients with submucosal cancer. Using 1:1 PS matching, a non-CR cohort of 22 matched pairs was established (Supplementary Table S2). The baseline characteristics of the patients and lesions were not significantly different between the two groups in the matched cohort. Kaplan–Meier analyses showed no significant differences in OS, DSS, or RFS between the two groups (Fig. 3). Among patients in the non-CR group after ESD, 11 (8%) underwent additional esophagectomy, and 17 (12.4%) received radiotherapy alone (Supplementary Table S3). Kaplan–Meier analysis showed no significant differences in OS and RFS between the non-CR ESD cohorts receiving additional esophagectomy and radiotherapy (Supplementary Fig. S2).

### Survival outcomes of the elderly cohort

**Figure 4:**
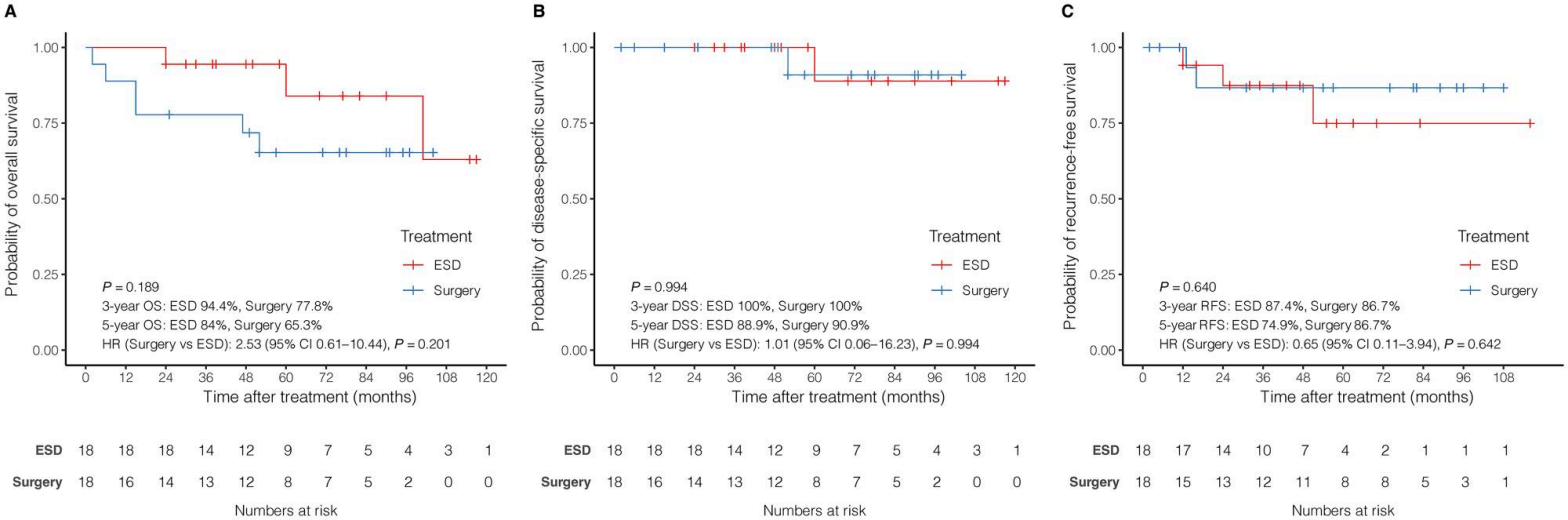
Kaplan-Meier survival curves of the ESD and esophagectomy groups in the elderly cohort. (A) OS of the elderly cohort; (B) DSS of the elderly cohort; and (C) RFS of the elderly cohort. OS, overall survival; DSS, disease-specific survival; RFS, recurrence-free survival; ESD, endoscopic submucosal dissection.

By using the same balancing method as in the main analysis, 1:1 PS matching was performed for 98 patients aged > 69 years. Eighteen matched pairs were included in this cohort. Supplementary Table S4 shows the baseline characteristics of the two subgroups. There were no significant differences in OS, DSS, or RFS between the two treatment groups in the elderly cohort (Fig. 4).

## Discussion

In this single-center, retrospective study with a PS-matched cohort, ESD and esophagectomy for SESCC showed similar long-term outcomes, suggesting that ESD may be an effective alternative to esophagectomy for patients with pT1 ESCC. ESD was associated with a shorter hospital stays and fewer adverse events. Similar outcomes were observed in patients with non-CR and in the elderly subgroup. In the analysis of preoperative EUS depth assessment, 27% of pT1a patients were over-staged by EUS. These findings highlight the effectiveness and safety of ESD for SESCC in real-world clinical practice, particularly given the limitations of preoperative depth assessment using EUS and the high risk of surgical complications. Given the limited accuracy of preoperative EUS in assessing invasion depth and the potential risks associated with esophagectomy, a staging ESD-first approach may represent a reasonable and pragmatic treatment strategy.

Preprocedural assessment of the cancer invasion depth is essential for estimating the risk of LN metastasis and guiding treatment strategies for SESCC. Previous studies have reported an overall accuracy of 63.2% (106/174) for EUS in differentiating invasion depth into three categories (EP/LPM, MM/SM1, and SM2) [4]. In a Korean study of 532 patients with ESCC, the accuracy of EUS in identifying cT1a was 82.3%; however, 39.5% of pTis–pT1a cases were over-staged as cT1b or deeper [22]. In the present study, although the accuracy of EUS may have been underestimated due to the exclusion of tumors invading the muscularis propria or regional LN, 27.0% of pT1a tumors (34/126) were assessed as cT1b or deeper (Supplementary Table S1), and most of these patients (32/34) underwent esophagectomy, resulting in two deaths from surgical complications (Supplementary Fig. S1). In the current clinical setting—where esophagectomy carries a high complication risk and preprocedural EUS has limited diagnostic accuracy, potentially leading to unnecessary surgery and unpredictable sequelae—these findings support the rationale for staging ESD in SESCC [13].

While ESD for SESCC provides accurate information on invasion depth through pathological evaluation, the implementation of ESD as a staging tool requires evidence that patients with non-CR after ESD can achieve a favorable prognosis and experience lower morbidity from ESD followed by additional treatment. In a prospective single-arm study of patients with T1b ESCC, the combination of endoscopic resection and CRT demonstrated comparable efficacy to surgery, without severe adverse events [14]. Kawaguchi *et al.* [23]. reported that, among patients with SESCC with pathologic T1b (±LVI) or m3 with vascular invasion treated with ESD followed by CRT, the 3-year OS (from initiation of CRT) was 90.0% compared with 63.2% in the definitive CRT group, and local recurrence was significantly lower (0% vs 19%, *P *= 0.029). Several retrospective studies have reported the efficacy and safety of ESD in patients with submucosal ESCC. A multicenter study from the Netherlands, which included 68 patients treated with ESD for early ESCC, found comparable long-term outcomes between the CR and non-CR groups. Additionally, outcomes in the non-CR group were not compromised by adjuvant treatment or observation, as the main cause of death was competing mortality rather than treatment complications or cancer recurrence [24]. Similarly, a study from Korea comparing primary esophagectomy with additional esophagectomy after ESD showed that additional surgery did not increase the risk of LN metastasis, and that the long-term outcomes were similar [25]. Zhang *et al.* [16] also reported no significant differences in the OS, DSS, or RFS between ESD and surgery in patients with T1b ESCC. Consistent with these studies, the subgroup analysis of the present study for the non-CR cohort showed no significant differences in OS, DSS, or RFS between the ESD and esophagectomy groups. Additionally, there were no significant differences in OS or RFS between the non-CR ESD cohorts who received additional esophagectomy and radiotherapy (Supplementary Fig. S2). However, further studies on the careful selection of additional treatments based on tumor characteristics and individual risk factors remain essential.

ESD and esophagectomy yielded comparable long-term clinical outcomes in patients with SESCC [8–11]. However, postoperative deterioration in quality of life remains a major concern, and esophagectomy carries a high risk of complications, with postoperative mortality rates exceeding 3% [8, 26–28]. Surgical treatment can pose a significant burden to elderly patients or those with comorbidities [29]. In contrast, ESD is a relatively safe and potentially curative treatment option, even in elderly patients with SESCC, and should be considered a primary treatment modality [19, 30]. In a study comparing endoscopic and surgical therapies for early ESCC in elderly patients, the 2-year survival rate after endoscopy was significantly higher than that after surgery [20]. Moreover, a multicenter retrospective study involving patients aged ≥75 years or with significant comorbidities found no significant difference in survival between patients who received additional treatment after noncurative ESD and those who underwent curative ESD [21]. Consistent with these reports, the present study also demonstrated the comparable efficacy and safety of ESD, particularly in the elderly subgroup. For patients unsuitable for esophagectomy or those who decline invasive treatments, ESD may serve as a minimally invasive and effective alternative.

This study had several limitations. First, this was a retrospective study, which inherently carries a risk of bias, despite PS matching. Although covariate balance was achieved, with absolute standardized mean differences below 0.1 and no statistically significant differences between the groups, the esophagectomy group tended to include tumors with larger size, more extensive circumferential involvement, and deeper invasion depth. In addition, the ESD group received more additional treatment than the surgery group, which might introduce treatment bias, and PS matching can only adjust for measured covariates. Notably, unmeasured confounding related to patients’ performance status, frailty, and detailed comorbidity burden beyond the CCI, as well as treatment selection factors in multidisciplinary discussions, may persist. Therefore, caution is required when interpreting the results of this study, and prospective studies are warranted to validate these results. Second, this was a single-center study, which may have limited the generalizability of the findings. Third, the overall sample size after matching was relatively small (*n *= 63), and the sample sizes of key subgroups, such as the non-CR (*n *= 22) and elderly cohorts (*n *= 18), were particularly limited. As a result, the estimates of OS, RFS, and DSS, which were not significantly different in the ESD group, should be interpreted with caution, as they may have been affected by imprecision owing to the small sample size. Further validation using larger, multicenter cohorts or public databases, ideally in the context of prospective studies incorporating biomarker analyses, is warranted to confirm our findings. Fourth, this study did not include molecular or genetic analyses (such as *TP53*, *NOTCH1*, *CDKN2A*, and *CCND1*) [31], which could provide additional insights into tumor biology and potential treatment responses.

In conclusion, ESD is a safe and effective alternative to esophagectomy for the treatment of SESCC, including in non-CR and elderly patients. With appropriate additional treatment and careful follow-up, the prognosis after ESD is comparable to that after primary esophagectomy. Given the limitations of preprocedural depth assessment and the high risk of complications associated with esophagectomy, staging ESD may represent a reasonable treatment option, particularly for elderly patients or those at high surgical risk.

## Supplementary Material

goag032_Supplementary_Data
