# Supplementary material for: Comparison of long-term outcomes between endoscopic submucosal dissection and esophagectomy for superficial esophageal squamous cell carcinoma
Source: Gastroenterol Rep (Oxf). 2026 Apr 19;14:goag032. doi: 10.1093/gastro/goag032 (PMC13092296; doi:10.1093/gastro/goag032)
Supplement: goag032_Supplementary_Data [file goag032_supplementary_data.docx]

**
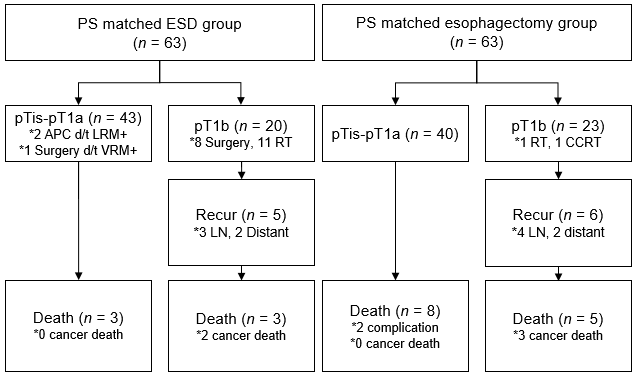
**

**Supplementary Figure S1. Brief summary of follow-up.**

PS, propensity score; ESD, endoscopic submucosal dissection; RT, radiation therapy; CCRT, concurrent chemoradiation; VRM, vertical resection margin; LN, lymph node.

**Supplementary Table S1. Degree of concordance between clinical stage assessed by preoperative endoscopic ultrasonography and pathologic stage reported by pathology**

| **Clinical stage** | **pT1a**  **(*N* = 126)** | **pT1b**  **(*N* = 77)** | **Total**  **(*N* = 203)** |
| --- | --- | --- | --- |
| **cT1a** | 92 (73.0%) | 23 (29.9%) | 115 (56.7%) |
| **cT1b** | 30 (23.8%) | 39 (50.6%) | 69 (34.0%) |
| **cT2 or deeper invasion** | 4 (3.2%) | 15 (19.5%) | 19 (9.3%) |

Note. Data are presented as numbers (%). Patients without preoperative invasion depth assessment were excluded from the study. The overall accuracy of distinguishing T1a from T1b was 71.2%.

Abbreviations: c, Clinical stage assessed by preoperative endoscopic ultrasonography; p, Pathological stage determined by histopathology.

**Supplementary Table S2. Baseline characteristics of the non-CR cohort**

| **Characteristic** |  | **ESD (*N* = 22)** | **Esophagectomy (*N* = 22)** | ***P* value** |
| --- | --- | --- | --- | --- |
| **Age, years** |  | 67.5 ± 8.3 | 74.4 ± 2.7 | 0.811 |
| **Male** |  | 21 (95.5%) | 17 (94.4%) | 1.000 |
| **CCI ≥ 2** |  | 5 (22.7%) | 3 (13.6%) | 0.696 |
| **Location**  **Cervical or upper thoracic**  **Middle thoracic**  **Lower thoracic or abdominal** |  | 2 (9.1%)  9 (40.9%)  11 (50.0%) | 1 (4.5%)  13 (59.1%)  8 (36.4%) | 0.464 |
| **Macroscopic type**  **Flat**  **Non-flat** |  | 17 (77.3%)  5 (22.7%) | 15 (68.2%)  7 (31.8%) | 0.735 |
| **Circumference of tumor**  **<1/4**  **1/4≤, <1/2**  **1/2≤, <3/4**  **3/4≤ or whole circumferential** |  | 8 (36.4%)  8 (36.4%)  3 (13.6%)  3 (13.6%) | 9 (40.9%)  9 (40.9%)  2 (9.1%)  2 (9.1%) | 0.915 |
| **Differentiation**  **Well**  **Moderate to poor** |  | 5 (22.7%)  17 (77.3%) | 5 (22.7%)  17 (77.3%) | 1.000 |
| **Tumor size, mm** |  | 22.6 ± 13.6 | 24.2 ± 13.7 | 0.702 |
| **Pathologic invasion depth**  **pTis**  **pT1a-LP**  **pT1a-MM**  **pT1b-SM1 (≤200 μm)**  **pT1b-SM2 (>200 μm)** |  | 0 (0.0%)  0 (0.0%)  1 (4.5%)  4 (18.2%)  17 (77.3%) | 0 (0.0%)  0 (0.0%)  1 (4.5%)  4 (18.2%)  17 (77.3%) | 1.000 |
| **Positive LV invasion** |  | 6 (27.3%) | 3 (13.6%) | 0.455 |
| **Resection margin**  **Positive lateral margin**  **Positive vertical margin** |  | 0 (0.0%)  4 (18.2%) | 1 (4.5%)  0 (0.0%) | 0.073 |
| **Non-curative resection**  **Positive vertical margin**  **Mucosal cancer with LV invasion**  **Submucosal cancer** |  | 4 (18.2%)  0 (0.0%)  18 (81.8%) | 0 (0.0%)  1 (4.5%)  21 (95.5%) | 0.073 |
| **Additional treatment**  **Observation**  **Surgery**  **Radiation**  **Chemoradiation** |  | 1 (4.5%)  8 (36.4%)  13 (59.1%)  0 (0.0%) | 19 (86.4%)  0 (0.0%)  1 (4.5%)  2 (9.1%) | <0.001 |

Note: Data are presented as mean ± standard deviation or number (%).

Abbreviations: ESD, endoscopic submucosal dissection; CCI, Charlson Comorbidity Index; LP, lamina propria; MM, muscularis mucosa; SM, submucosa; LV, lymphovascular.

**Supplementary Table S3. Baseline characteristics of the non-curative resection after endoscopic submucosal dissection cohort**

| **Characteristic** |  | **Additional esophagectomy (*N* = 11)** | **Additional radiotherapy**  **(*N* = 17)** | ***P* value** |
| --- | --- | --- | --- | --- |
| **Age, years** |  | 61.9 ± 5.6 | 69.2 ± 7.4 | 0.010 |
| **Male** |  | 11 (100.0%) | 16 (94.1%) | 1.000 |
| **CCI ≥ 2** |  | 1 (9.1%) | 5 (29.4%) | 0.419 |
| **Location**  **Cervical or upper thoracic**  **Middle thoracic**  **Lower thoracic or abdominal** |  | 1 (9.1%)  5 (45.5%)  5 (45.5%) | 1 (5.9%)  8 (47.1%)  8 (47.1%) | 0.949 |
| **Macroscopic type**  **Flat**  **Non-flat** |  | 9 (81.8%)  2 (18.2%) | 14 (82.4%)  3 (17.6%) | 1.000 |
| **Circumference of tumor**  **<1/4**  **1/4≤, <1/2**  **1/2≤, <3/4**  **3/4≤ or whole circumferential** |  | 3 (27.3%)  5 (45.5%)  2 (18.2%)  1 (9.1%) | 9 (52.9%)  4 (23.5%)  2 (11.8%)  2 (11.8%) | 0.520 |
| **Differentiation**  **Well**  **Moderate to poor** |  | 1 (9.1%)  10 (90.9%) | 8 (47.1%)  9 (52.9%) | 0.092 |
| **Tumor size, mm** |  | 21.4 ± 12.3 | 19.4 ± 11.4 | 0.663 |
| **Pathologic invasion depth**  **pTis**  **pT1a-LP**  **pT1a-MM**  **pT1b-SM1 (≤200 μm)**  **pT1b-SM2 (>200 μm)** |  | 0 (0.0%)  0 (0.0%)  2 (18.2%)  1 (9.1%)  8 (72.7%) | 0 (0.0%)  1 (5.9%)  1 (5.9%)  5 (29.4%)  10 (58.8%) | 0.349 |
| **Positive LV invasion** |  | 6 (54.5%) | 6 (35.3%) | 0.539 |
| **Resection margin**  **Positive lateral margin**  **Positive vertical margin** |  | 0 (0.0%)  2 (18.2%) | 0 (0.0%)  2 (11.8%) | 1.000 |
| **Non-curative resection**  **Positive vertical margin**  **Mucosal cancer with LV invasion**  **Submucosal cancer** |  | 2 (18.2%)  1 (9.1%)  8 (72.7%) | 2 (11.8%)  2 (11.8%)  13 (76.5%) | 0.883 |

Note: Data are presented as mean ± standard deviation or number (%).

Abbreviations: ESD, endoscopic submucosal dissection; CCI, Charlson Comorbidity Index; LP, lamina propria; MM, muscularis mucosa; SM, submucosa; LV, lymphovascular.

**Supplementary Table S4. Baseline characteristics of the elderly cohort**

| **Characteristic** |  | **ESD (*N* = 18)** | **Esophagectomy (*N* = 18)** | ***P* value** |
| --- | --- | --- | --- | --- |
| **Age, years** |  | 75.0 ± 4.3 | 74.4 ± 2.7 | 0.642 |
| **Male** |  | 17 (94.4%) | 17 (94.4%) | 1.000 |
| **CCI ≥ 2** |  | 1 (5.6%) | 1 (5.6%) | 1.000 |
| **Location**  **Cervical or upper thoracic**  **Middle thoracic**  **Lower thoracic or abdominal** |  | 1 (5.6%)  5 (27.8%)  12 (66.7%) | 1 (5.6%)  6 (33.3%)  11 (61.1%) | 0.935 |
| **Macroscopic type**  **Flat**  **Non-flat** |  | 14 (77.8%)  4 (22.2%) | 16 (88.9%)  2 (11.1%) | 0.655 |
| **Circumference of tumor**  **<1/4**  **1/4≤, <1/2**  **1/2≤, <3/4**  **3/4≤ or whole circumferential** |  | 6 (33.3%)  7 (38.9%)  2 (11.1%)  3 (16.7%) | 7 (38.9%)  7 (38.9%)  2 (11.1%)  2 (11.1%) | 0.964 |
| **Differentiation**  **Well**  **Moderate to poor** |  | 9 (50.0%)  9 (50.0%) | 10 (55.6%)  8 (44.4%) | 1.000 |
| **Tumor size, mm** |  | 23.4 ± 12.6 | 24.5 ± 12.7 | 0.794 |
| **Pathologic invasion depth**  **pTis**  **pT1a-LP**  **pT1a-MM**  **pT1b-SM1 (≤200 μm)**  **pT1b-SM2 (>200 μm)** |  | 5 (27.8%)  3 (16.7%)  1 (5.6%)  1 (5.6%)  8 (44.4%) | 7 (38.9%)  3 (16.7%)  2 (11.1%)  1 (5.6%)  5 (27.8%) | 0.851 |
| **Positive LV invasion** |  | 3 (16.7%) | 2 (11.1%) | 1.000 |
| **Resection margin**  **Positive lateral margin**  **Positive vertical margin** |  | 0 (0.0%)  2 (11.1%) | 0 (0.0%)  0 (0.0%) | 0.467 |
| **Non-curative resection**  **Positive vertical margin**  **Submucosal cancer** |  | 2 (11.1%)  7 (38.9%) | 0 (0.0%)  6 (33.3%) | 0.286 |
| **Additional treatment**  **Observation**  **Endoscopy**  **Surgery**  **Radiation** |  | 9 (50.0%)  1 (5.6%)  1 (5.6%)  7 (38.9%) | 18 (100.0%)  0 (0.0%)  0 (0.0%)  0 (0.0%) | 0.007 |

Note: Data are presented as mean ± standard deviation or number (%).

Abbreviations: ESD, endoscopic submucosal dissection; CCI, Charlson Comorbidity Index; LP, lamina propria; MM, muscularis mucosa; SM, submucosa; LV, lymphovascular.

**
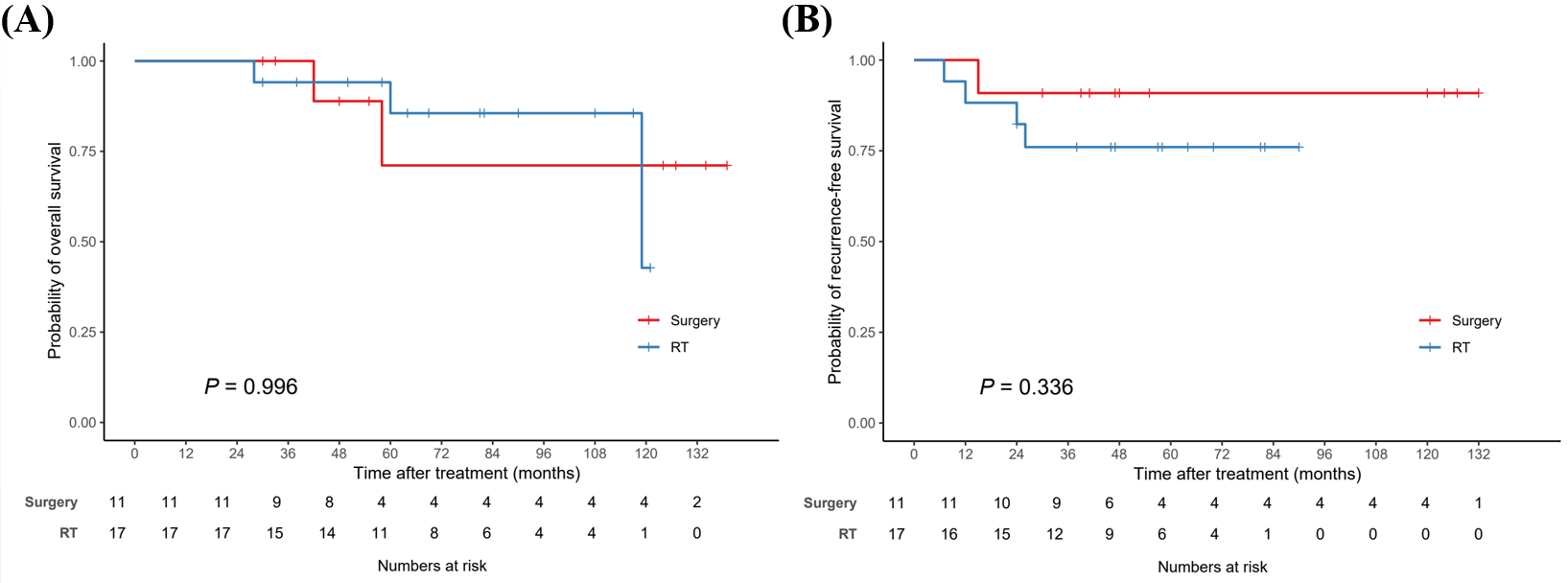
 Supplementary Figure S2. Kaplan-Meier survival curves according to additional treatment modality (surgery versus radiation therapy) in patients with non-curative resection after ESD. A, Overall survival; B, Recurrence-free survival.**

PS, propensity score; ESD, endoscopic submucosal dissection; RT, radiation therapy; CCRT, concurrent chemoradiation; VRM, vertical resection margin; LN, lymph node.
